# Supplementary material for: Evolutionary Divergence and Radula Diversification in Two Ecomorphs from an Adaptive Radiation of Freshwater Snails
Source: Genes (Basel). 2022 Jun 8;13(6):1029. doi: 10.3390/genes13061029 (PMC9222583; doi:10.3390/genes13061029)
Supplement: Supplementary file 1 [file genes-13-01029-s001.zip › genes-1748794-supplementary.pdf]

Supplement

Evolutionary Divergence and Radula Diversification in Two Ecomorphs from an Adaptive Radiation of Freshwater Snails

Leon Hilgers [1,2,3,*], Stefanie Hartmann [2], Jobst Pfaender [4], Nora Lentge-Maaß [3], Ristiyanti M. Marwoto [5], Thomas von Rintelen [3,†] and Michael Hofreiter [2,†]

1 LOEWE Centre for Translational Biodiversity Genomics (TBG), 60325 Frankfurt, Germany
2 Institute of Biochemistry and Biology, Universität Potsdam, 14476 Potsdam, Germany; stefanie.hartmann@uni-potsdam.de (S.H.); michael.hofreiter@uni-potsdam.de (M.H.)
3 Museum für Naturkunde, 10115 Berlin, Germany; nora.lentge-maass@mfn.berlin (N.L.-M.); thomas.vonrintelen@mfn.berlin (T.v.R.)
4 Naturkundemuseum Potsdam, 14467 Potsdam, Germany; jobst.pfaender@rathaus.potsdam.de
5 Museum Zoologicum Bogoriense, Research Centre for Biosystematics and Evolution, National Research and Innovation Agency, Cibinong 16912, Indonesia; ristiyanti456@gmail.com
* Correspondence: leon.hilgers@senckenberg.de
† Thomas von Rintelen & Michael Hofreiter are shared senior authors.

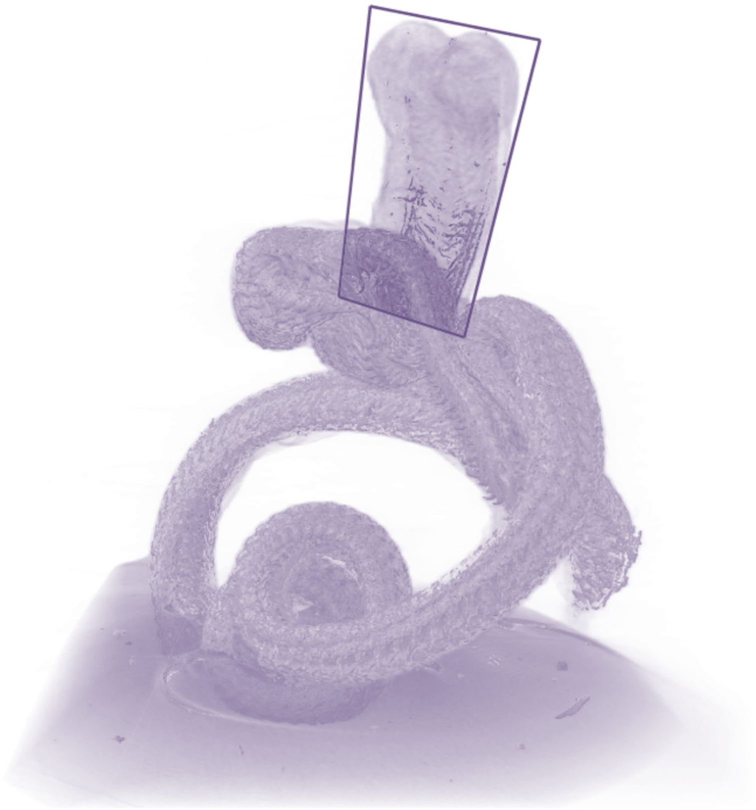

Figure S1. Micro-CT image of a dissected *Tylomelania* radula. The sampled radula forming tissue, where the radula is continuously secreted, is indicated by a box. Color differences represent differing densities and show starting tooth hardening (darker areas).

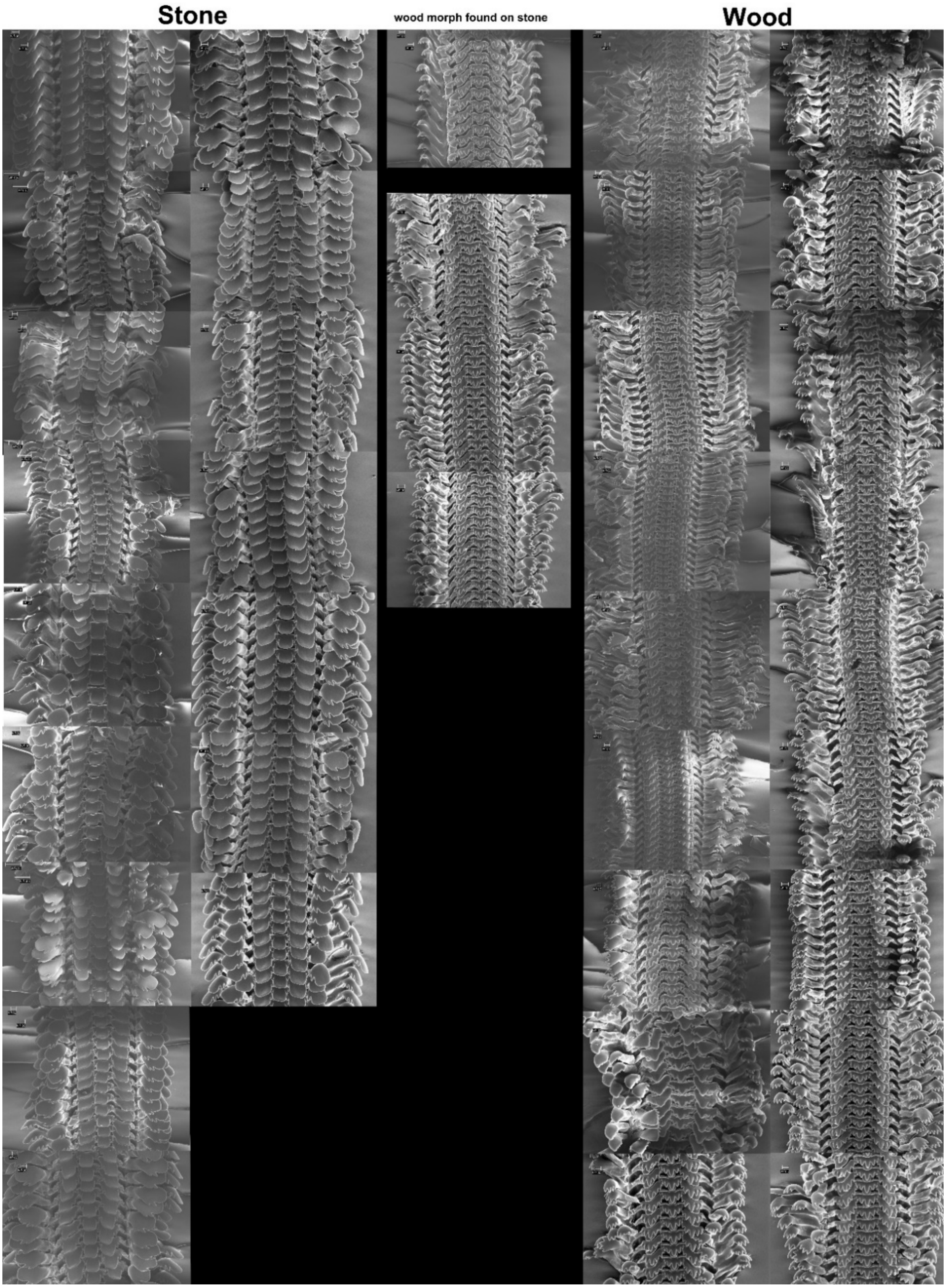

Figure S2. Overview of all radulae of individuals that were randomly collected from wood and rock substrates for morphological analyses. SEM images of radulae from *T. sarasinorum* rock morphs collected from rock (left), wood morph collected on wood (right) and wood morph collected on rock (middle) are shown. All individuals are shown, but one rock individual that was collected could not be used for further morphological analyses, because of insufficient resolution of morphological features required for measurements.

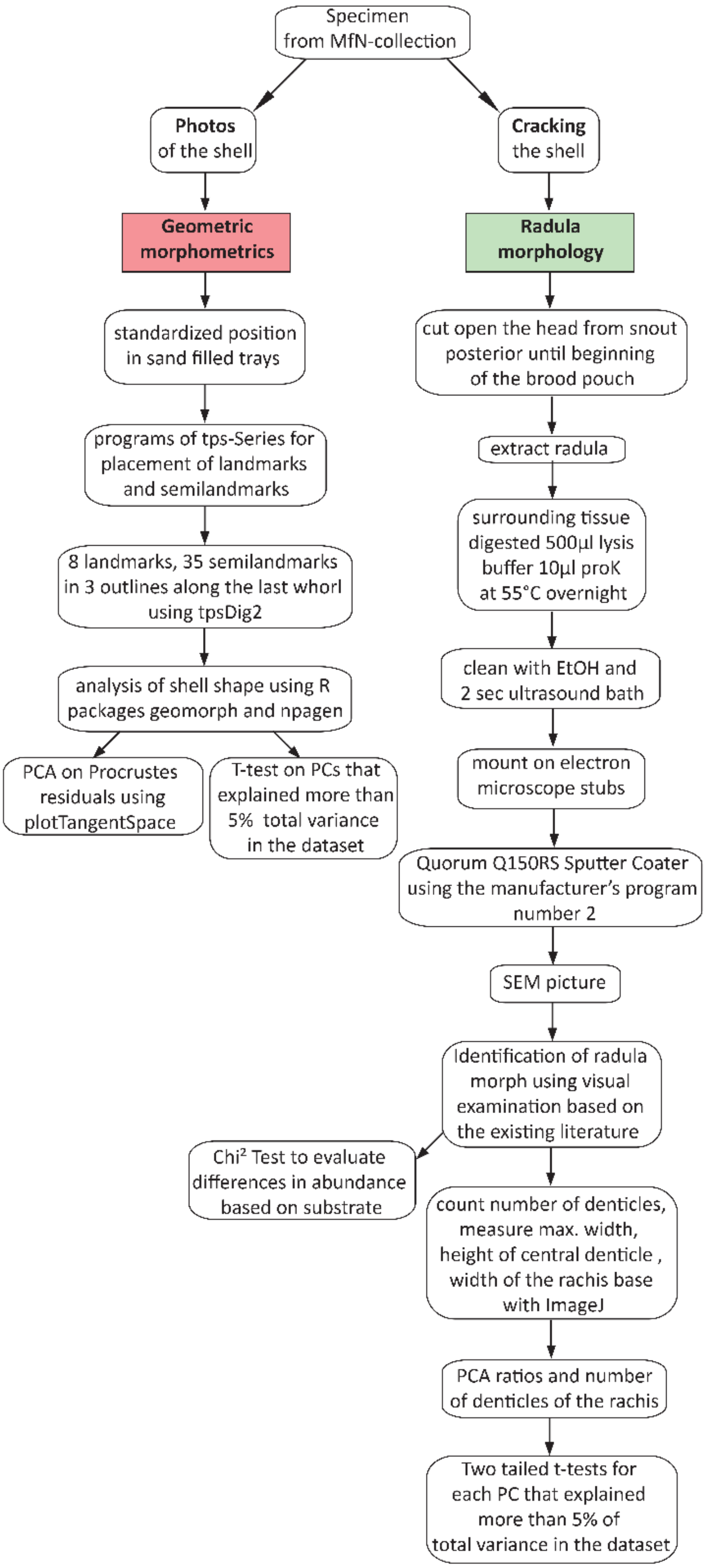

Figure S3. Flowchart illustrating the sampling and analyses of the morphological dataset.

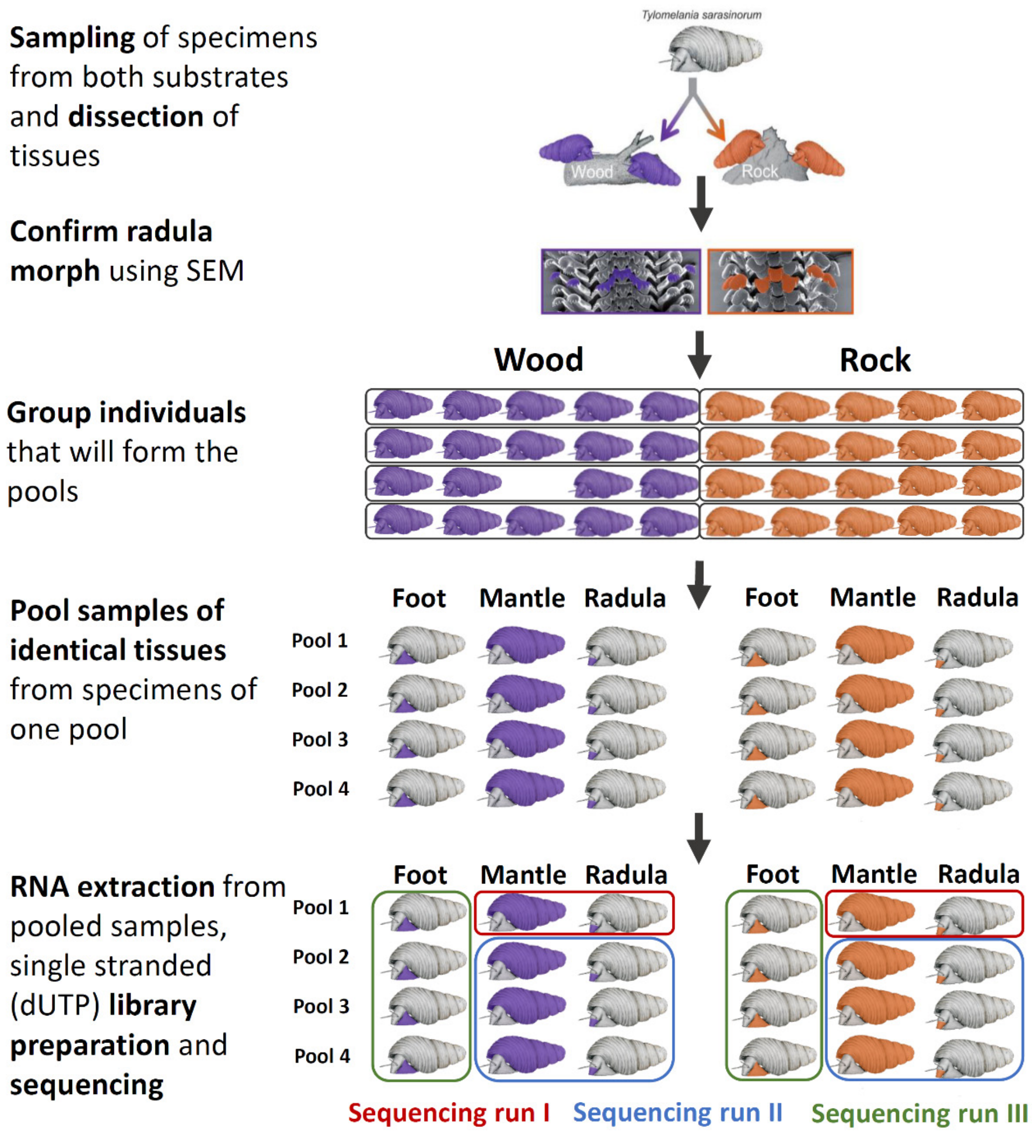

Figure S4. Schematic overview of specimen sampling, pooling and how samples were combined in sequencing runs to generate the molecular data presented in this manuscript.

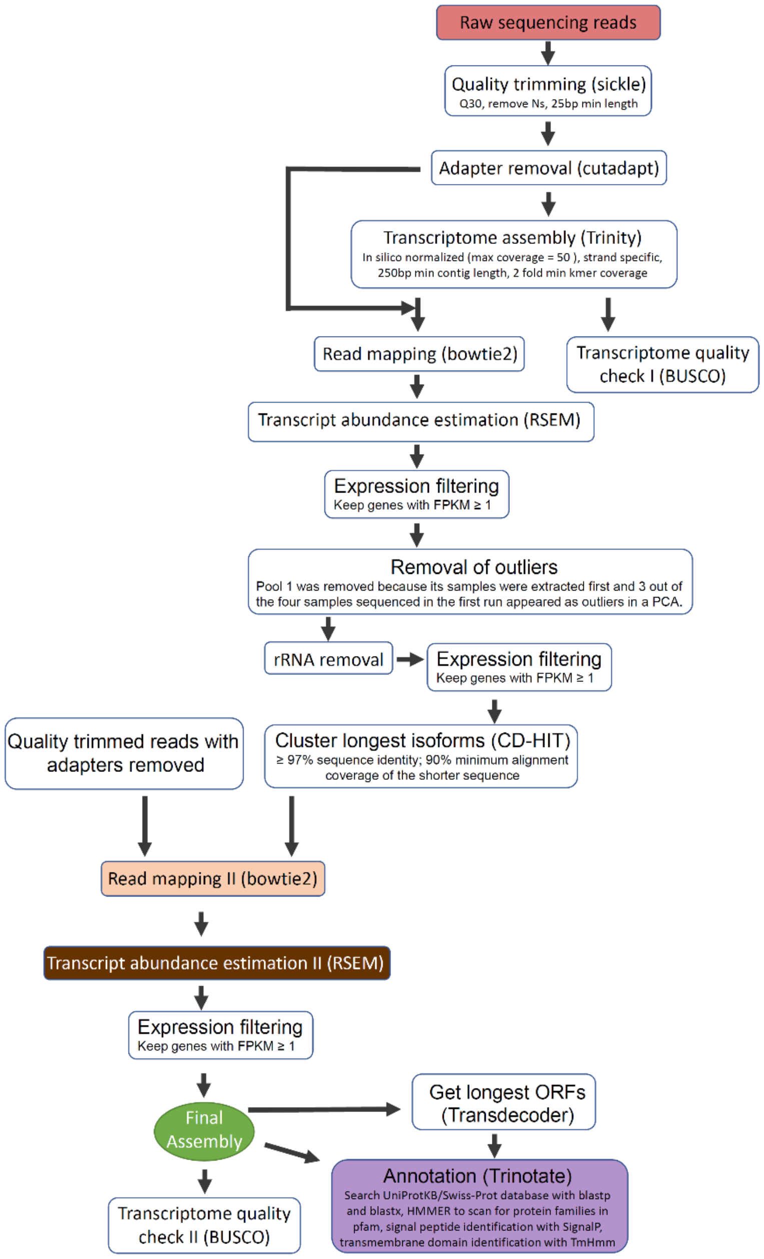

Figure S5. Schematic overview of molecular analyses from quality trimming of raw sequencing reads to the final assembly and its annotation. Boxes in the flowchart indicate how data was treated in each step and arrows show in which follow up analyses the generated data was used. Colored ellipses represent key datasets within this manuscript.

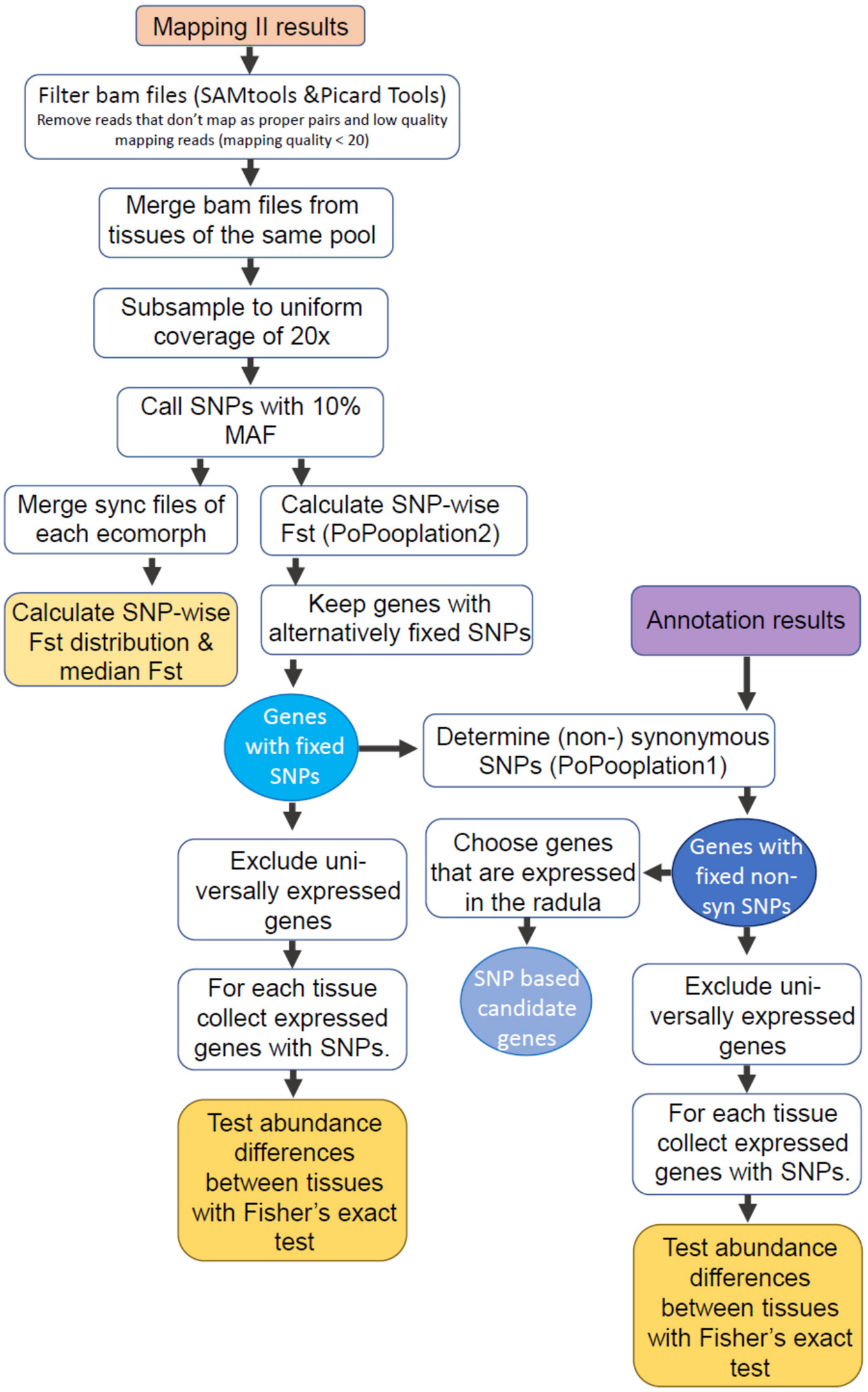

Figure S6. Schematic overview of SNP data and population analyses. Boxes in the flowchart indicate how data was treated in each step and arrows show in which follow up analyses the generated data was used. Colored ellipses represent key datasets within this manuscript.

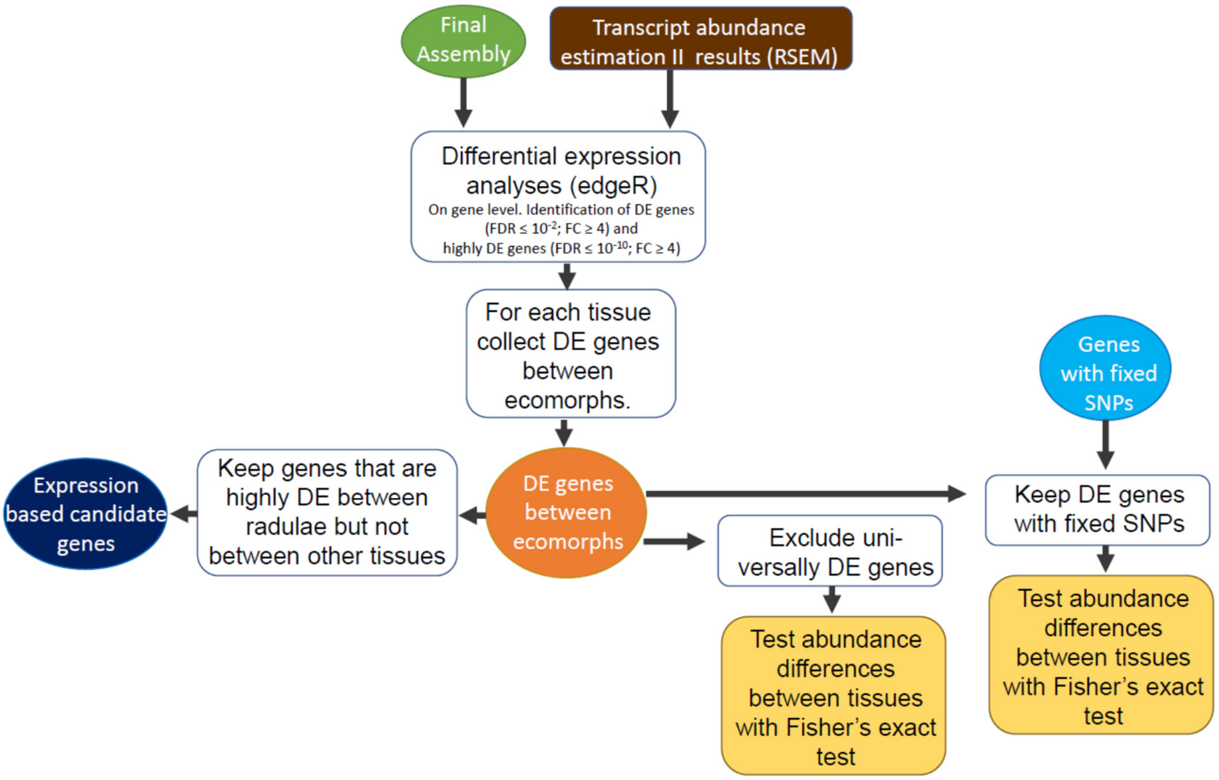

Figure S7. Schematic overview of gene expression analyses. Boxes in the flowchart indicate how data was treated in each step and arrows show in which follow up analyses the generated data was used. Colored ellipses represent key datasets within this manuscript. Analyses that were carried out with both DE genes and highly DE genes are only shown once for DE genes to increase readability.

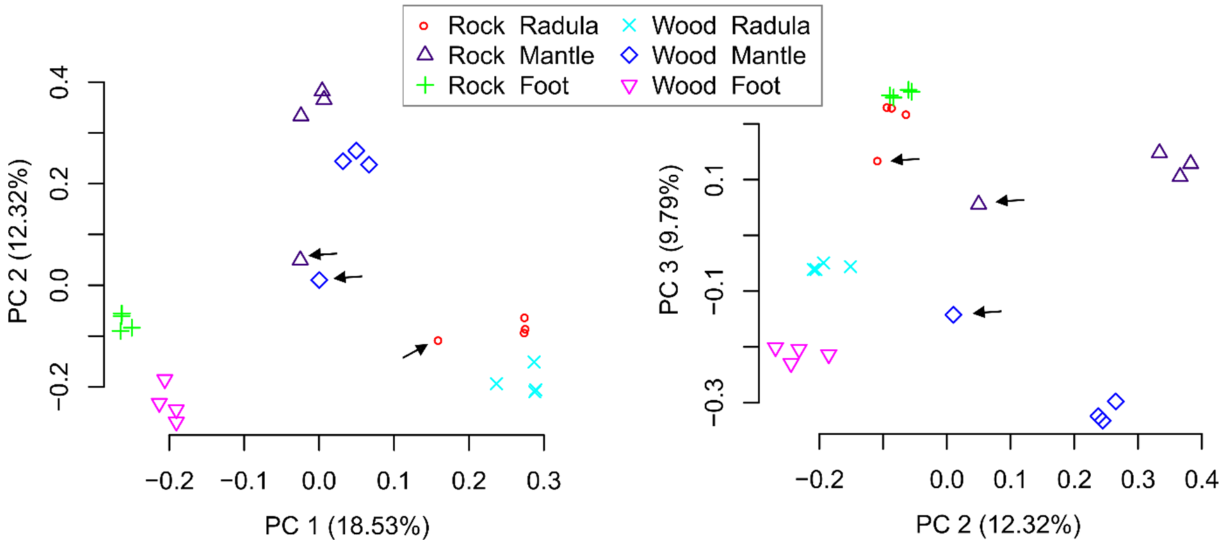

Figure S8. Principal component analyses of gene expression before filtering, including outlier samples. Tissue samples that led us to the decision to exclude samples of pool1 of both ecomorphs are marked with black arrows.

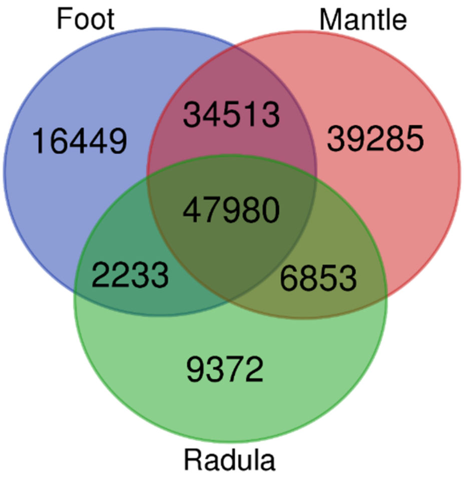

Figure S9. Venn graph illustrating the number of uniquely and jointly expressed genes across the three tissues. A gene was considered to be expressed in a certain tissue if it was expressed with FPMK ≥ 1 in at least one biological replicate of at least one of the two ecomorphs.

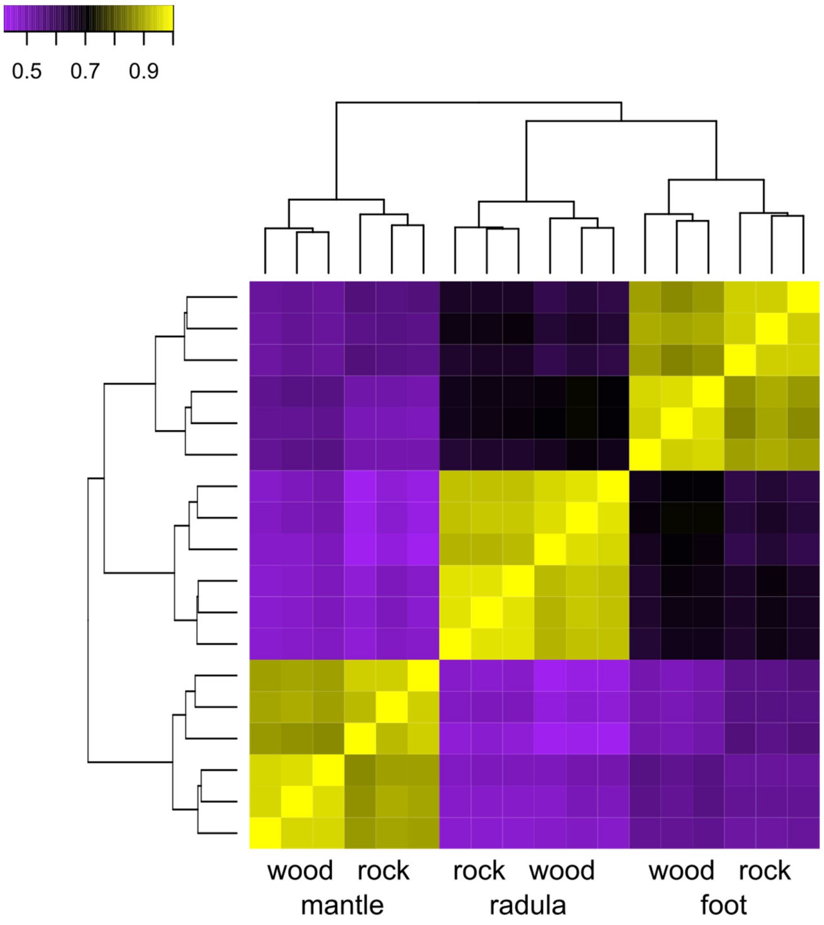

Figure S10. Hierarchically clustered Spearman correlation matrix of gene expression (log_2 transformed counts per million mapped reads) with the same number of expressed genes in all tissues. The number of expressed genes was equalized across tissues by setting expression of a random set of expressed genes to 0 in all samples of a both ecomorphs. Samples with more similar gene expression cluster together in the matrix and the hierarchical clustering tree (left and top). Color gradient from purple to yellow shows increasing correlation in gene expression between samples. Compared to Figure 4b, expression between radula ecomorphs appears less similar and overall divergence in gene expression is very similar across all tissues. Hence, the lower number of expressed genes in the radula increases the pairwise correlation between all radula tissues and thereby underestimates overall divergence between radula transcriptomes compared to mantle and foot.

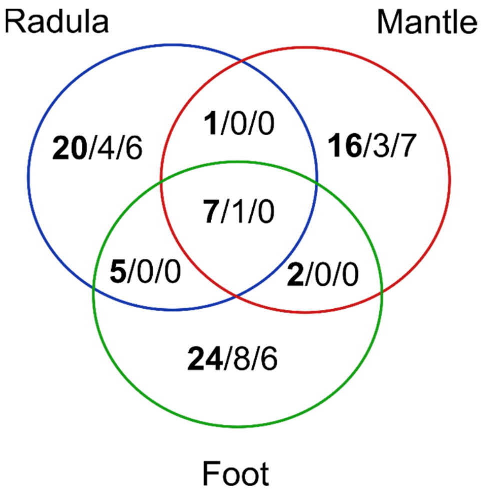

Figure S11: Venn graph illustrating the presence of alternatively fixed SNPs in transcripts of genes that are also differentially expressed (FDR ≤ 10^{-5}) between at least one pair of identical tissues of both ecomorphs. The total number of SNPs in highly DE genes is shown first and in bold, followed by the number of synonymous and non-synonymous SNPs in these genes.

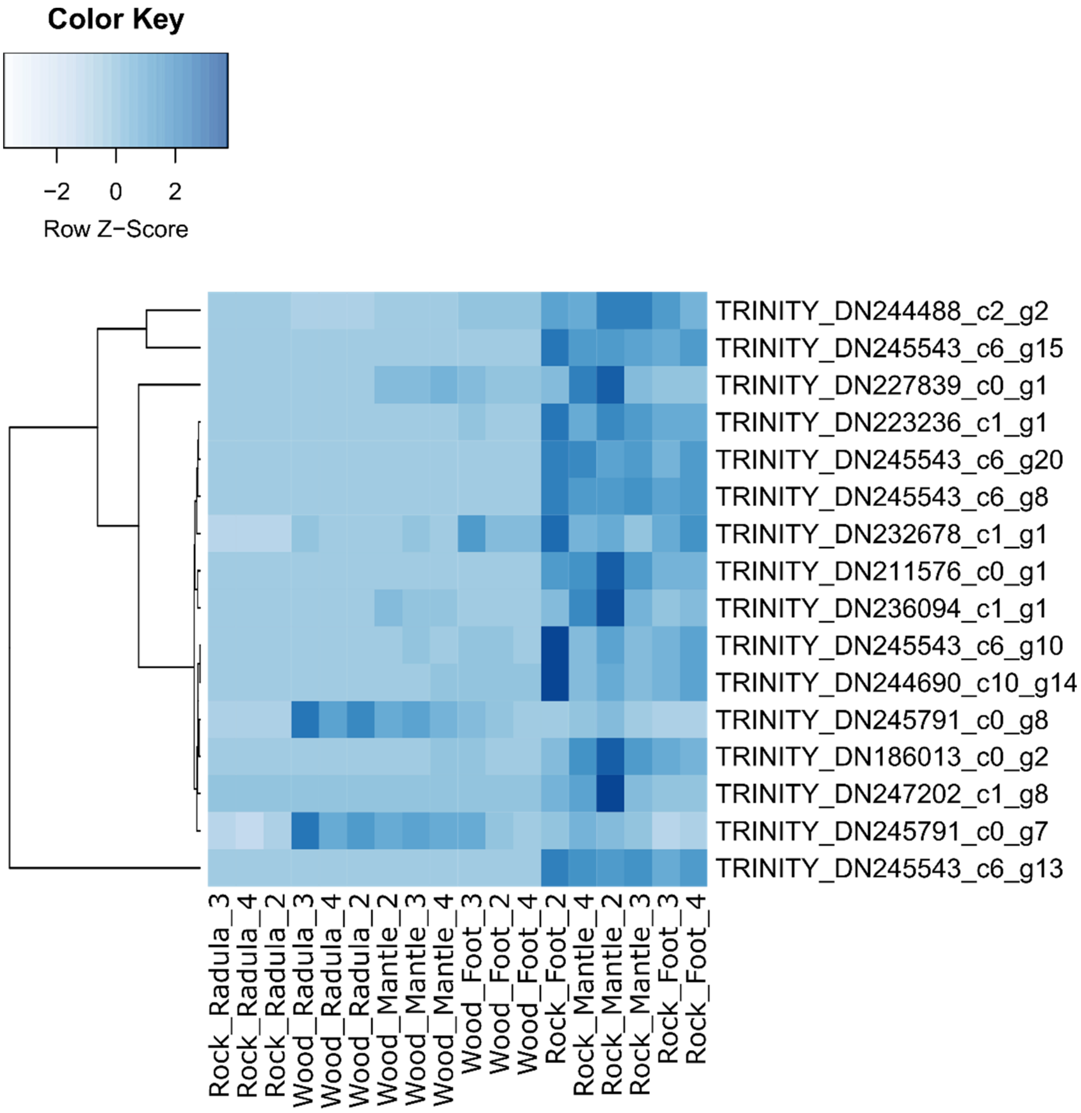

Figure S12. Gene expression of differentially expressed genes with the MF tetrapyrrole binding (GO:0046906). Hierarchical clustering heatmap of tetrapyrrole binding genes that were enriched among differentially expressed genes between ecomorphs. Samples and genes with similar expression cluster together. Heatmap is colored according to the row-wise z-score, which means

that genes overexpressed in a certain sample relative to the other samples are colored dark blue, while underexpressed genes are colored light blue in the heatmap.

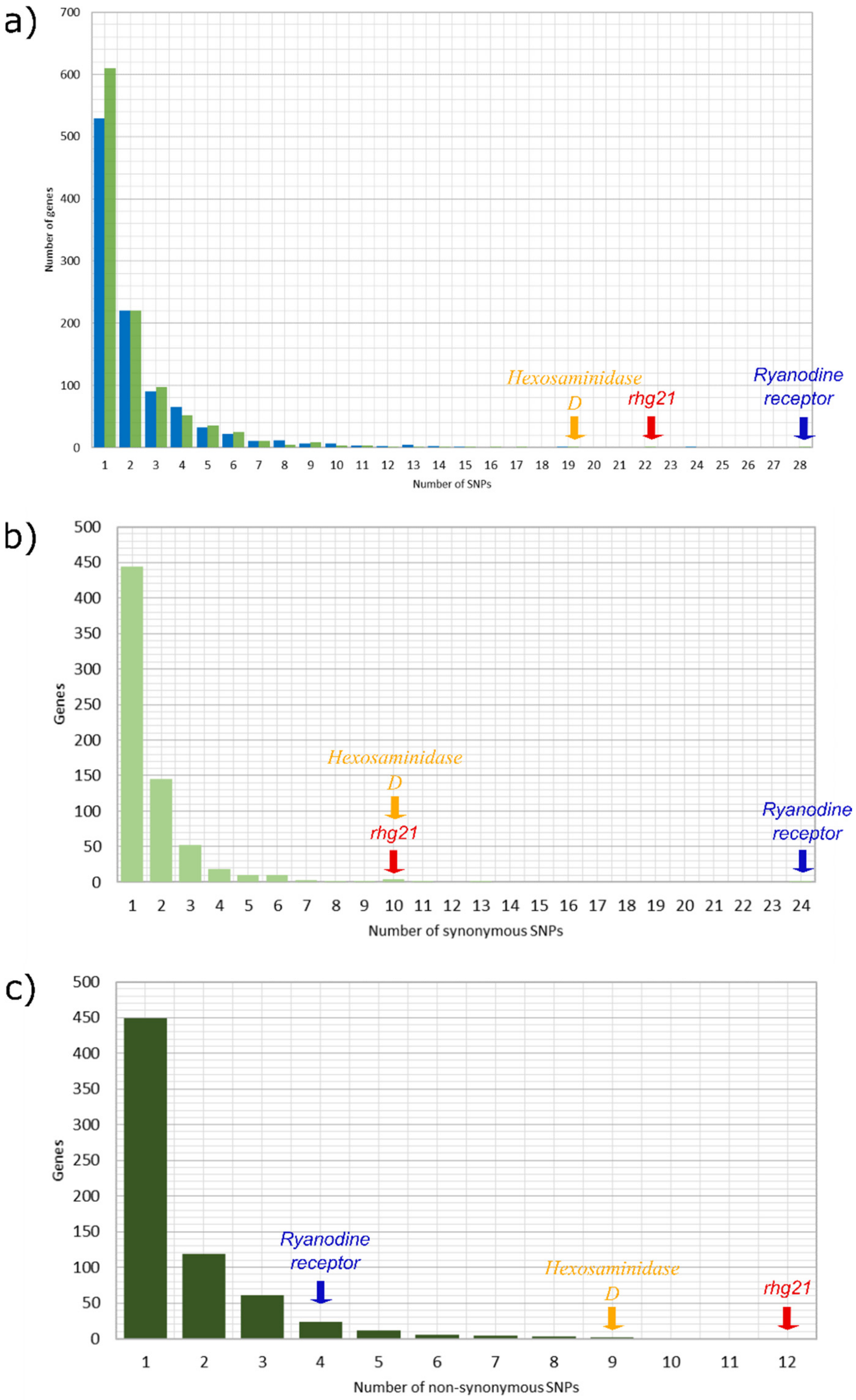

Figure S13. Distributions of alternatively fixed SNP numbers per transcript. a) Numbers of alternatively fixed SNPs per transcript inside (green) and outside (blue) of ORFs and the number of b) synonymous and c) non-synonymous alternatively fixed SNPs per gene are shown. Colored arrows indicate the three genes with the highest number of SNPs inside their ORFs in a) and show the number of synonymous and non-synonymous SNPs in b) and c), respectively. Only transcripts that carry at least one alternatively fixed SNP are included in this figure.

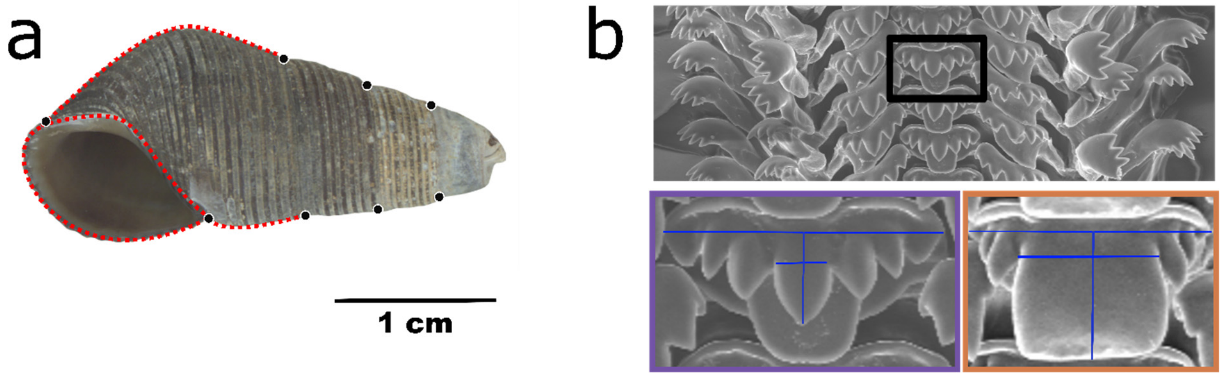

Figure S14. Morphological characterization of *T. sarasinorum* shell and radula. a) Landmarks (black) and semi-landmarks (red) were positioned on digital photographs of *T. sarasinorum* shells from individuals collected from wood and rock substrates at Loeha Island. Shell morphology was measured with eight landmarks, i.e., one on the most apical end of the aperture, one on the opposite end of the aperture, at the visual intersection of the aperture with the outside of the first whorl and each one at either side of the shell at the suture of the first and the second whorl, the second and the third whorl, and the fourth and the fifth whorl. Four semilandmarks were used to characterize the aperture, outer lip and shape of the first whorl. These consisted of 10 sliding landmarks with the exception of the shortest stretch from the top of the aperture to the top of the first whorl (most bottom right semilandmark). b) Length measurements (blue lines) of the rachis (black box) and its central rachis denticle were used to characterize radula shape of *T. sarasinorum* wood (purple) and rock (orange) ecomorphs.

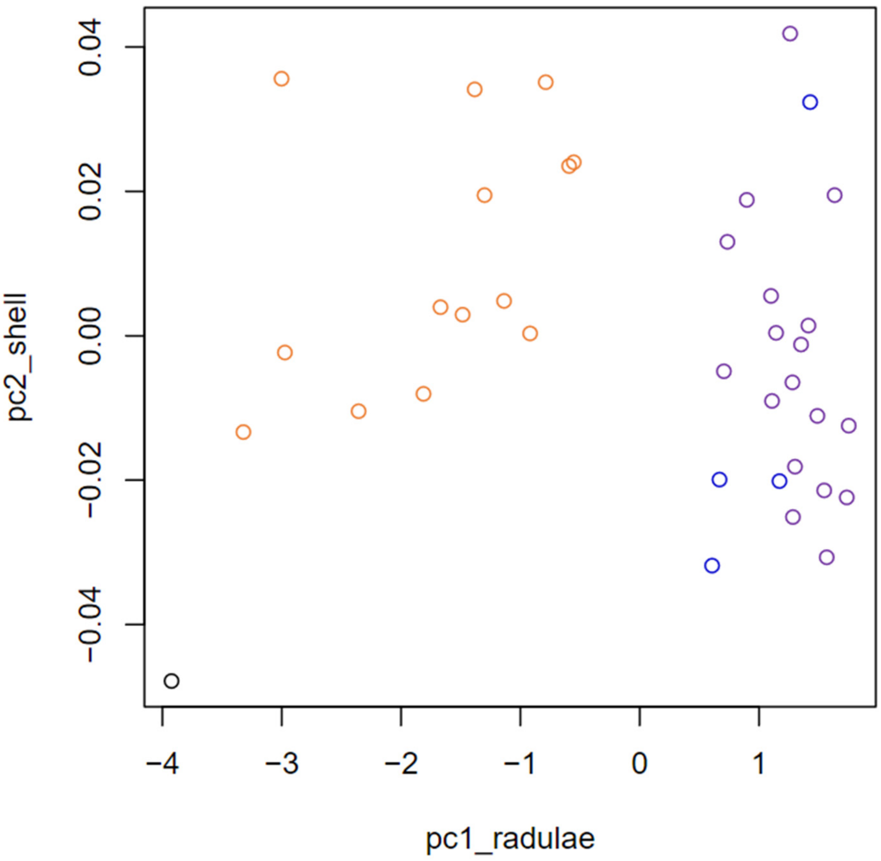

Figure S15. Scatterplot based on the two principal components of shell and radula shape that differed significantly between wood (purple) and rock (orange) ecomorphs. Specimens of the wood ecomorph collected on rock substrate are illustrated in blue. This plot differs from Figure 2b in that the number of denticles on the rachis was excluded from the radula PCA presented in the main text. While the explained variance of PC1 of radula shape increases from 86.3% to 93.66% this plot shows that all main results are independent of whether count data are included in this analysis.

Table S1. Principle components of shell shape and the proportion of variance explained.

	Standard deviation	Proportion of variance	Cumulative variance
**PC1**	0.02157	0.42405	0.42405
**PC2**	0.01138	0.11802	0.54207
**PC3**	0.01043	0.09905	0.64112
**PC4**	0.008205	0.061340	0.702460
**PC5**	0.00757	0.05221	0.75467
**PC6**	0.006746	0.041470	0.796140
**PC7**	0.006332	0.036540	0.832680
**PC8**	0.005894	0.031650	0.864330
**PC9**	0.004971	0.022520	0.886850
**PC10**	0.004803	0.021020	0.907870
**PC11**	0.003991	0.014510	0.922380
**PC12**	0.003837	0.013420	0.935800
**PC13**	0.003287	0.009850	0.945650
**PC14**	0.003046	0.008450	0.954100
**PC15**	0.002852	0.007410	0.961520

Table S2. T-test results for shell size and PCs of shape as well as PC1 of radula shape. Significant differences between ecomorphs are indicated by bold p-values.

Trait		t	df	*p*
Shell	PC1	0.95	33.47	0.351
Shell	PC2	5.67	33.01	**0.000**
Shell	PC3	0.54	28.48	0.590
Shell	PC4	0.29	34.99	0.774
Shell	PC5	-0.004	32.75	0.997
Shell	PC6	-1.11	32.19	0.275
Shell	Size	-1.30	33.31	0.203
Radulae	PC1	-5.22	93.18	**0.000**

Table S3. Assembly statistics of the raw and filtered assembly.

	Trinity genes	GC (in %)	'gene' N50[b]	Complete[a] (in %)	Duplicated[a] (in %)
**Raw assembly**	478 661	45.2	613	89	9.4
**Filtered assembly**	156 685	44.9	1 229	89	7.5

[a] According to BUSCO; [b] based on the longest isoform per gene.

Table S4. Enriched gene ontologies in genes with alternatively fixed non-synonymous SNPs and differentially expressed genes between identical tissues of both ecomorphs.

Gene set	GO	Description	Log10 (p-value)
DE genes between ecomorphs	**BP**		
	-	-	-
	**CC**		
	-	-	-
	**MF**		
	GO:0004497	monooxygenase activity	-4.97
	GO:0016712	oxidoreductase activity acting on paired donors with incorporation or reduction of molecular oxygen reduced flavin or flavoprotein as one donor and incorporation of one atom of oxygen	-4.83
	GO:0016705	oxidoreductase activity acting on paired donors with incorporation or reduction of molecular oxygen	-4.18
	GO:0005506	iron ion binding	-4.18
	GO:0046906	tetrapyrrole binding	-3.64
	GO:0016491	oxidoreductase activity	-2.70
	GO:0030246	carbohydrate binding	-2.70
	GO:0020037	heme binding	-2.48
Genes with alternatively fixed non-synonymous SNPs	**BP**		
	GO:0008150	Biological process	-2.35
	**CC**		
	GO:0005575	Cellular component	-2.77
	**MF**		
	-	-	-

Table S5. Number of paired-end reads before and after quality filtering.

Sample	# mio raw pe reads	# mio quality filtered pe reads
Pool1 Wood Mantle	52.63	45.47
Pool1 Wood Radula	136.52	121.5
Pool1 Wood Foot	44.13	38.51
Pool2 Wood Mantle	37.11	32.82
Pool2 Wood Radula	29.14	26.23
Pool2 Wood Foot	41.69	36.1
Pool3 Wood Mantle	32.5	28.31
Pool3 Wood Radula	41.36	36.96
Pool3 Wood Foot	47.33	41.52
Pool4 Wood Mantle	34.33	29.69
Pool4 Wood Radula	27.21	24.26
Pool4 Wood Foot	37.29	31.92
Pool1 Rock Mantle	54.19	47.07
Pool1 Rock Radula	65.64	58.16
Pool1 Rock Foot	45.07	39.15
Pool2 Rock Mantle	37.07	33.1
Pool2 Rock Radula	38.73	34.45
Pool2 Rock Foot	39.21	34.3
Pool3 Rock Mantle	30.62	27.24
Pool3 Rock Radula	39.71	35.49
Pool3 Rock Foot	38.67	33.72
Pool4 Rock Mantle	37.6	33.29
Pool4 Rock Radula	38.98	34.8
Pool4 Rock Foot	42.46	37.09
**Sum**	1069.19	941.15

Table S6. Mapping rates before the last expression-based filtering step (mapped with bowtie2).

Sample	Rock	Wood
**Pool2_Radula**	83,52%	84.68%
**Pool3_Radula**	82,33%	83.24%
**Pool4_Radula**	82,14%	82.51%
**Pool2_Mantle**	78,27%	80.59%
**Pool3_Mantle**	77,81%	80.29%
**Pool4_Mantle**	78,10%	79.11%
**Pool2_Foot**	81,32%	84.24%
**Pool3_Foot**	82,54%	84.17%
**Pool4_Foot**	82,08%	84.41%
**Median**	82,08%	83.24%
